# Association of Thallium with Diabetes Risk among Patients with Hearing Loss: Result from NHANES 2013 to 2018

**DOI:** 10.1097/MD.0000000000037317

**Published:** 2024-03-01

**Authors:** Jing Li, Zhi-Gang Lai, Xiao-Hua Huang

**Affiliations:** a Otolaryngology department, Longyan First Affiliated Hospital of Fujian Medical University, Longyan 364000, Fujian, China.

**Keywords:** diabetes risk, hearing loss, model construction, NHANES database, Thallium

## Abstract

To evaluate the correlation between thallium and diabetes risk among participants with hearing loss. This retrospective cohort study extracted related data such as demographic characteristics, lifestyle factors, and laboratory findings from the National Health and Nutrition Examination Survey (NHANES) database (2013–2018). Logistic regression analysis and interaction analysis were adopted to analyze the correlation between thallium and diabetes risk among patients with hearing loss. Then, the restricted cubic spline was employed to assess the nonlinear relationship between thallium and diabetes risk. The receiver operating characteristic curve and decision curve analysis were used to assess the predictive values of 3 multivariate models with or without thallium for diabetes risk. The Delong test was adopted to assess the significant change of the area under the curves (AUCs) upon thallium addition. A total of 425 participants with hearing loss were enrolled in the study: without diabetes group (*n* = 316) and diabetes group (*n* = 109). Patients with hearing loss in the diabetes group had significantly lower thallium (*P* < .05). The thallium was an independent predictor for diabetes risk after adjusting various covariates (*P* < .05). The restricted cubic spline (RCS) result showed that there was a linear correlation between thallium and diabetes risk (*P* nonlinear > .05). Finally, the receiver operating characteristic and decision curve analysis results revealed that adding thallium to the models slightly increased the performance in predicting diabetes risk but without significance in AUC change. Thallium was an independent predictor of diabetes risk among patients with hearing loss. The addition of thallium might help improve the predictive ability of models for risk reclassification. However, the conclusions should be verified in our cohort in the future due to the limitations inherent in the NHANES database.

## 1. Introduction

Hearing loss is the fourth leading contributor to years lived with disability, which represents a significant global health burden^[1]^ About 1 billion young adults are at risk of hearing loss and there are 466 million individuals with disabling hearing loss worldwide reported by the World Health Organization.^[2]^ Multiple factors such as aging, noise exposure, infection, and congenital may result in hearing loss.^[1]^ It is evident that hearing loss presents close correlations with dementia, cognition, and risk for hospitalization, unemployment, and depression.^[3–7]^ Diabetes as a chronic multisystem disease is characterized by elevated glucose levels in the blood and urine because of inadequate production or use of insulin. Approximately 460 million adults have diabetes with a global incidence of 6.4%, which is expected to reach 7.7% by 2030.^[8]^ Diabetes is related to neuropathy, retinopathy, kidney disease, and cardiovascular disease.^[2]^ Many patients with diabetes have a probability of developing hearing loss since high glucose levels may damage the vessels of the nerves and stria vascularis.^[9]^ However, lack of research focus on the diabetes risk among patients with hearing loss. Clinically, hearing loss refers to a pure tone average >25 dB; however, hearing conditions vary widely across grades, and mild hearing loss may be reversible after self-healing or modifying incentives.^[10]^ Thus, we adopted data on hearing conditions with severe hearing difficulty as study populations.

Thallium is an extremely toxic heavy metal, independently discovered by Crookes and Lamy in 1861 from the dust of sulfuric acid burning equipment.^[11]^ It is widely used in industrial activities such as electronic equipment, semiconductors, pesticides, and rodenticides.^[12]^ Besides, anthropogenic activities also lead to increased concentrations of thallium in the environment, causing adverse health impacts on humans.^[13,14]^ Since thallium is odorless, tasteless, and water-soluble, humans are under low-dose thallium exposure in their daily lives by eating contaminated food and water or the inhaling contaminated air.^[15,16]^ Thallium induces the symptoms with 3 main categories: motor function impairment (muscle weakness and paralysis of the legs, etc.), gastrointestinal damage (vomiting and nausea, etc.), and nerve damage (anorexia nervosa and numbness, etc.).^[17,18]^ Previous studies have demonstrated that exposure to heavy metals was a risk factor for metabolic abnormalities including hypertension, obesity, metabolic syndrome, and diabetes.^[19,20]^ Cadmium, lead, arsenic, and mercury had positive correlations with nonalcoholic fatty liver disease.^[21–24]^ However, little is known about the association of thallium with diabetes risk among individuals with hearing loss.

Accordingly, this study was conducted to analyze the correlation between thallium and diabetes risk among participants with hearing loss by using the data from the National Health and Nutrition Examination Survey (NHANES) years 2013 to 2018.

## 2. Materials and methods

### 2.1. Study population

The NHANES is a program of studies designed to assess the health and nutritional status of the population in the United States, which is implemented every 2 years (https://www.cdc.gov/nchs/nhanes/index.htm). The NHANES database can be acquired publicly from the National Center for Health Statistics of the Centers for Disease Control and Prevention (CDC). The survey protocol was approved by the National Center for Health Statistics Institutional Review Board, and written informed consent was obtained from all the participants. The NHANES interview includes demographic, socioeconomic, dietary, and health-related questions. The examination component consists of medical, dental, and physiological measurements and laboratory tests. Our study extracted data from the 2013 to 2018 cycles. Totally 28,232 samples were obtained from 2013 to 2018. Exclusion criteria: without diabetes information (*n* = 529); without thallium information (*n* = 19,357); 7921 subjects having no hearing loss. Finally, 425 participants with hearing loss were enrolled in the study.

### 2.2. Assessment of hearing loss and diabetes

Hearing loss refers to serious difficulty hearing in this study. Trained interviewers provided survey participants with a household questionnaire about disability. Hearing loss is determined by affirmative responses to the question: Are you deaf or do you have serious difficulty hearing?^[25]^ Diabetes is determined according to the answer to the following question: Other than during pregnancy, have you/has SP ever been told by a doctor or health professional that you have/he/she/SP has diabetes or sugar diabetes?

### 2.3. Covariates

We extracted the following data from the NHANES database. Demographic variables included age, gender, race, and poverty. The federal poverty level (FPL) referring to the ratio of family income to poverty, was used to assess the family’s income conditions. Body measure index (BMI) was collected from the “Body measures” in the “Examination data.” Laboratory data including thallium, albumin, alkaline phosphatase (AKP), aspartate aminotransferase (AST), alanine aminotransferase (ALT), blood urea nitrogen, total calcium (TC), cholesterol, creatine phosphokinase (CPK), creatinine, triglycerides, and uric acid were collected from the “Laboratory data.” The alcohol user was regarded as a participant who had drunk alcohol over the past 12 months. A smoker was regarded as a participant who had smoked at least 100 cigarettes in their entire life. Health insurance was evaluated by the following question: Are you/is SP covered by health insurance or some other kind of health care plan? Hypertension was determined by the answer to “Have you/has SP ever been told by a doctor or other health professional that you/s/he had hypertension, also called high blood pressure?” Detailed information can be accessed on the official website (https://www.cdc.gov/nchs/nhanes/index.htm).

### 2.4. Statistical analysis

Categorical variables were represented by count (percent) and group comparisons were evaluated by the Chi-square test or Fisher test. The Shapiro–Wilk test was adopted to analyze the data distribution of continuous variables, which were presented as mean ± standard deviation for normally distributed data and median (interquartile range) for nonnormally distributed data. The comparisons for continuous variables were analyzed by Student *t* test (normal distribution) or Mann–Whitney *U* test (skewed distribution). The results showed that all the continuous variables were normally distributed except for thallium. Considering the skewed distribution of thallium, Log2-transformation was conducted to facilitate interpretation.

Logistic regression analysis was employed to evaluate the correlation of thallium as a continuous variable and as quartiles with diabetes risk among participants with hearing loss. Univariate model; multivariate Model 1: demographic characteristics and BMI were adjusted; multivariate Model 2: the variables in Model 1 in addition to lifestyle factors, health insurance, and hypertension were adjusted; multivariate Model 3: adjusting the variables in Model 2 as well as laboratory findings. Then, the association of thallium with diabetes risk was conducted stratified by age, gender, BMI, alcohol use, smoking, and hypertension. Further, the restricted cubic spline was adopted to evaluate the nonlinear correlation between thallium and diabetes risk. Interaction analysis was performed to obtain the interactors for this correlation.

Moreover, the area under the curve (AUC) of the receiver operating characteristic (ROC) curves were calculated to evaluate the predictive ability of each model, and the Delong test was employed to examine the change of AUCs upon thallium addition. Finally, ROC analysis and decision curve analysis were employed to compare the efficacy of models in predicting diabetes risk.

All data analyses were carried out by SPSS software version 23.0 and R software version 4.2.2. *P* < .05 was considered statistically significant.

## 3. Results

### 3.1. Patient characteristics

Totally 425 patients with hearing loss were enrolled in the study and they were categorized into without diabetes group (*n* = 316) and with diabetes group (*n* = 109). Patient characteristics are shown in Table 1. Patients with diabetes are more likely to be older, nonalcoholic users, suffer from hypertension and have higher BMI (all *P* < .05). Besides, those with diabetes tend to exhibit significantly elevated levels of AST, ALT, and triglycerides but have lower cholesterol and thallium (all *P* < .05). However, no obvious differences in gender, race, income level, health insurance, smoker, albumin, AKP, blood urea nitrogen, TC, CPK, creatinine, and uric acid were observed between the 2 groups (all *P* > .05).

**Table 1:** Characteristics of the patients with hearing loss from NHANES 2013–2018.

Variables	Without diabetes (*n* = 316)	With diabetes (*n* = 109)	*P* value
Age, years	65.000 (49.000, 78.000)	70.000 (62.000, 78.000)	0.003
Gender, %			
Male	194 (61.392)	75 (68.807)	0.166
Female	122 (38.608)	34 (31.193)	
BMI, kg/m 2	27.854 ± 6.051	32.130 ± 6.532	<0.001
Race, %			
Hispanic	81 (25.633)	32 (29.358)	0.830
White	160 (50.633)	55 (50.459)	
Black	40 (12.658)	12 (11.009)	
Other races	35 (11.076)	10 (9.174)	
Income level, %			
<100% FPL	68 (23.529)	27 (28.125)	0.820
100–199% FPI	101 (34.948)	32 (33.333)	
200–499% FPI	81 (28.028)	26 (27.083)	
≥500% FPI	39 (13.495)	11 (11.459)	
Health insurance, %			
No	39 (12.342)	12 (11.009)	0.712
Yes	277 (87.658)	97 (88.991)	
Alcohol user, %			
No	35 (25.547)	28 (45.161)	0.006
Yes	102 (74.453)	34 (54.839)	
Smoker, %			
No	123 (41.695)	46 (42.202)	0.927
Yes	172 (58.305)	63 (57.798)	
Hypertension, %			
No	154 (51.852)	28 (25.688)	<0.001
Yes	143 (48.148)	81 (74.312)	
Albumin, g/L	41.363 ± 3.290	41.356 ± 3.687	0.985
Alkaline phosphatase, IU/L	76.014 ± 27.385	77.346 ± 23.624	0.660
Aspartate aminotransferase, U/L	23.195 ± 9.109	26.000 ± 10.223	0.015
Alanine aminotransferase, U/L	21.452 ± 12.546	24.548 ± 12.626	0.032
Blood urea nitrogen, mg/dL	16.658 ± 6.515	17.817 ± 7.675	0.139
Total calcium, mg/dL	9.356 ± 0.378	9.365 ± 0.401	0.837
Cholesterol, mg/dL	191.390 ± 40.116	177.250 ± 48.619	0.009
Creatine Phosphokinase, IU/L	142.705 ± 223.169	161.577 ± 252.846	0.477
Creatinine, mg/dL	0.987 ± 0.304	1.058 ± 0.519	0.096
Triglycerides, mg/dL	156.476 ± 119.958	196.452 ± 133.396	0.005
Uric acid, mg/dL	5.639 ± 1.425	5.707 ± 1.531	0.686
Thallium, ug/L	0.150 [0.090, 0.230]	0.110 [0.080, 0.160]	<0.001

### 3.2. Association of thallium with diabetes risk among patients with hearing loss

We first treated thallium as a continuous variable to analyze its correlation with diabetes risk among patients with hearing loss. In the univariate logistic regression analysis, as the thallium level increased, the risk of developing diabetes was decreased (*P* < .01). After adjusting age, gender, race, income level, and BMI in multivariate model 1, thallium was remarkably related to a decreased diabetes risk with an odds ratio (OR) of 0.301. After adjusting covariates in model 1 as well as alcohol user, hypertension, health insurance, and smoking, the significance still existed in the correlation between thallium and diabetes risk (OR = 0.119) (*P* < .01). After adjusting all covariates in multivariate model 3, thallium elevation was still an independent predictor for reduced diabetes risk (OR = 0.125) (*P* < .01). Then, we explored the association of thallium with diabetes risk when thallium was coded into 4 quartiles. Compared with patients in Q1, those in Q4 had a notably lower risk of developing diabetes in all models (all *P* < .05) except in multivariate model 2 (*P* > .05) (Table 2). Moreover, patients with elevated thallium levels tended to have lower odds of diabetes (*P* for trend < 0.05) (Table 2).

**Table 2:** The association of Thallium with diabetes risk among patients with hearing loss.

	Univariate model		Multivariate model 1		Multivariate model 2		Multivariate model 3	
	OR (95% CI)	*P* value	OR (95% CI)	*P* value	OR (95% CI)	*P* value	OR (95% CI)	*P* value
Log Thallium	0.277 (0.132–0.579)	0.001	0.301 (0.125–0.725)	0.007	0.119 (0.030–0.467)	0.002	0.125 (0.027–0.576)	0.008
Thallium quartiles								
Q1	Reference		Reference		Reference		Reference	
Q2	1.129 (0.673–1.895)	0.646	1.189 (0.648–2.179)	0.576	1.334 (0.540–3.294)	0.532	1.945 (0.704–5.372)	0.199
Q3	0.525 (0.266–1.034)	0.062	0.509 (0.232–1.121)	0.094	0.676 (0.229–1.992)	0.477	0.927 (0.266–3.237)	0.906
Q4	0.390 (0.180–0.843)	0.017	0.530 (0.221–1.273)	0.156	0.110 (0.019–0.633)	0.013	0.088 (0.011–0.677)	0.020
*P* for trend	0.004			0.044		0.017		0.044

95% CI = 95% confidence interval, OR = odds ratio

**Figure 1.**
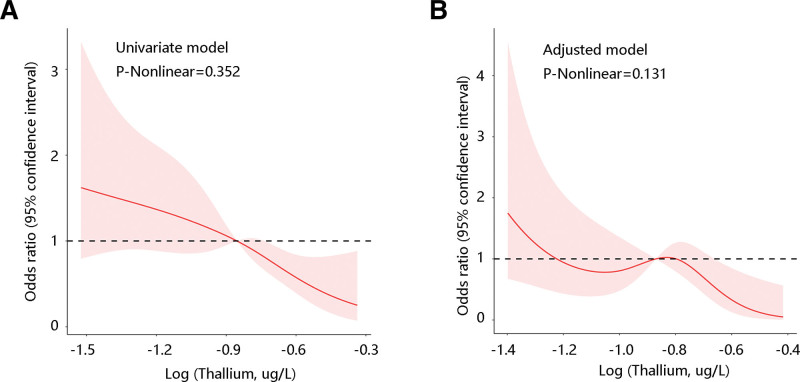
Restricted cubic spline (RCS) analysis for the nonlinear relationship between thallium and diabetes risk among patients with hearing loss. (A) Univariate model. (B) Adjusted model.

To further screen out the low-risk population of diabetes, subgroup analysis was performed. High thallium levels were closely linked to low diabetes risk among patients with hearing loss in spite of age, alcohol user, and smoker (all *P* < .05). Additionally, a significant negative relationship was found between thallium and diabetes risk among males, those with BMI ≥ 30, and with hypertension (all *P* < .05). However, this correlation was not significant in females, those with BMI < 25 group, 25 ≤ BMI < 30 group, and no hypertension group (all *P* > .05) (Table 3). Subsequently, we observed a linear relationship between the thallium and diabetes risk in the univariate model and adjusted model (*P* nonlinear > .05) (Fig. 1A, B).

**Table 3:** Subgroup analysis of the association of Thallium with diabetes risk among patients with hearing loss.

Subgroups	Odds ratio (95% confidence interval)	*P* value	*P* for interaction
Age			
<65	0.236 (0.072–0.770)	0.017	0.789
≥65	0.369 (0.139–0.979)	0.045	
Gender			
Male	0.235 (0.091–0.606)	0.003	0.548
Female	0.314 (0.092–1.074)	0.065	
BMI			
<25	0.558 (0.065–4.787)	0.595	0.291
25 ≤ BMI < 30	0.470 (0.098–2.253)	0.345	
≥ 30	0.150 (0.051–0.435)	<0.001	
Alcohol user			
No	0.080 (0.010–0.646)	0.018	0.177
Yes	0.171 (0.043–0.677)	0.012	
Smoker			
No	0.292 (0.092–0.924)	0.036	0.061
Yes	0.293 (0.108–0.792)	0.016	
Hypertension			
No	0.349 (0.088–1.377)	0.133	0.751
Yes	0.304 (0.119–0.776)	0.013	

BMI = body mass index

### 3.3. Addictive value of thallium in diabetes risk prediction

**Figure 2.**
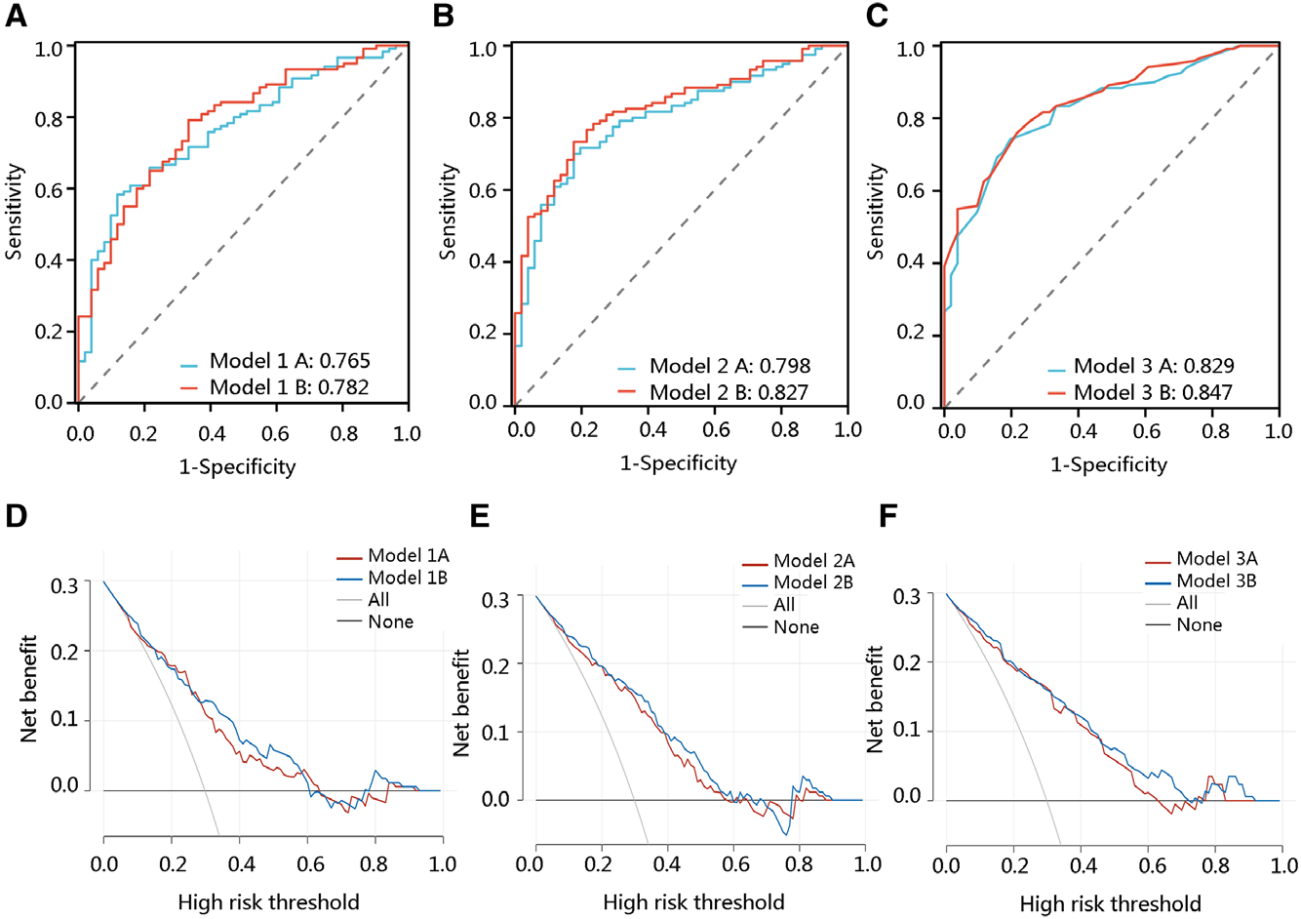
The predictive value of the models with or without thallium for diabetes risk among patients with hearing loss. Receiver operating characteristic curve analysis showing the predictive value of (A) model 1, (B) model 2, and (C) model 3. Model 1A: Model 1 without thallium; Model 1B: Model 1with thallium; Model 2A: Model 2 without thallium; Model 2B: Model 2 with thallium; Model 3A: Model 3 without thallium; Model 3B: Model 3 with thallium.

The predictive value of each multivariate model was determined. The AUCs for the base model 1, model 2, and model 3 were 0.765, 0.798, and 0.828, respectively. After adding thallium to the models, the AUCs were changed to 0.782, 0.827, and 0.849, respectively, but without significant improvement (all *P* > .05) (Table 4) (Figs. 2A–C). The decision curve analysis results further confirmed that the clinical efficacy of models had s slight increase after adding thallium (Fig. 2D–F). Although no significant improvement in model performance, thallium alone had a satisfactory performance in predicting diabetes risk among patients with hearing loss with an AUC of 0.718 (Figure S1, Supplemental Digital Content, http://links.lww.com/MD/L793).

**Table 4:** Performance metrics of multivariate models with and without Thallium to predict diabetes among patients with hearing loss.

	AUC (95% confidence interval)	*P* value for Δ AUC
Model 1 without Thallium	0.765 (0.691–0.839)	0.440
Model 1 with Thallium	0.782 (0.709–0.854)	
Model 2 without Thallium	0.798 (0.730–0.867)	0.093
Model 2 with Thallium	0.827 (0.765–0.890)	
Model 3 without Thallium	0.828 (0.765–0.891)	0.202
Model 3 with Thallium	0.849 (0.791–0.906)	

Model 1: adjusting demographic characteristics and BMI; Model 2: the variables in Model 1 in addition to lifestyle factors, health insurance, and hypertension were adjusted; Model 3: the variables in Model 2 as well as laboratory findings were adjusted

AUC = are under the curve

## 4. Discussion

This retrospective study found that thallium was significantly associated with diabetes risk among participants with hearing loss, particularly in males, with a BMI ≥ 30, and with hypertension. This correlation remains significant regardless of age, alcohol user, and smoker. Besides, restricted cubic spline revealed a linear relation between thallium and diabetes risk in both the univariate model and the adjusted model. ROC analysis results showed that the addition of thallium to the multivariate models contributed to a slight increase of AUC in predicting diabetes risk but without significant change.

Industrial activities result in the release of approximately 5000 tons of thallium into the environment each year.^[12]^ Increased levels of thallium in farm animals, fruits, vegetables, and tap water may upregulated the risk of long-term low-dose exposure to thallium in the general population. Thallium was detectable in urine samples and the concentration of thallium in pregnant women in Australia is 0.29 μg/L, and 0.17 μg/L in both pregnant women and adults in the United States.^[26–28]^ In this study, 0.15 and 0.11 μg/L were detected in urine samples of participants without and with diabetes group, respectively. As a heavy metal, thallium has a negative impact on human beings’ health. Higher thallium concentrations during pregnancy were related to an increased risk of preterm birth, low birth weight, and premature rupture of membranes.^[29,30]^ In addition, Qi et al^[31]^ reported that pregnant women with thallium exposure might result in stunted growth in early childhood. Zhang et al^[32]^ exhibited that pregnant women with the highest thallium level were at higher risk of developing gestational diabetes mellitus. Nevertheless, the correlation between thallium and diabetes risk among patients with hearing loss has not been clarified.

Thallium exposure could bring burdens to the stomach, leading to various gastrointestinal symptoms including anorexia and decreased appetite.^[18]^ Li et al^[33]^ reported that the food intake of mice was inhibited upon thallium exposure. Besides, the immune system including immune stimulation and immune inhibition can be affected upon exposure to heavy metals.^[34]^ Immunoglobulin levels would be reduced in the blood after being exposed to lead.^[35]^ B cell apoptosis would increase and its development would be impaired after thallium exposure.^[36]^ Thallium exposure induces reactive oxygen species excess in cells and promotes oxidative stress.^[37]^ Then, Na+/K+-ATPase activity inhibition, and isolated mitochondrial swelling lead to damaged mitochondrial function, which is irreparable.^[38]^ Next, impaired mitochondrial structure and function may block the electron transport chain, leading to ATP depletion and inducing mitophagy, thereby reducing the mitochondrial DNA copy number (mtDNAcn).^[39–41]^ To maintain optimal physiological functions, mtDNAcn remains within a relatively stable range.^[42]^ As a surrogate marker for mitochondrial function and intracellular oxidative stress, mtDNAcn is implicated in the pathogenesis of multiple diseases.^[43]^ High mtDNAcn level was associated with a lower risk of depression and mortality.^[44]^ Besides, individuals with elevated levels of mtDNAcn are at decreased risk of possessing cardiovascular disease.^[45]^ Based on these findings, the authors speculated that thallium elevation results in mtDNAcn reduction, which might cause adverse impacts on health conditions. Surprisingly, we found that higher thallium levels were independently linked to lower diabetes risk among participants with hearing loss. However, the addition of thallium to 3 models led to an insignificant increase in predictive performance. Given that its lower levels were detected in both groups, thallium may not play a decisive role but a reference value in predicting diabetes risk.

For strengths, this is the first study to analyze the correlation between thallium and diabetes risk among patients with hearing loss. Besides, we adopted 3 different models with or without thallium to compare its value in predicting diabetes risk. Although the cross-sectional study designs can only infer correlation, but causality, this study provides a reference for clinical practice in the future. For limitations, this study may have potential biases in data collection or limitations inherent in the NHANES database. These findings especially the clinical relevance of adding thallium to the models should be validated in our cohort in the future. Further research could explore the biological mechanisms underlying the observed correlation between thallium and diabetes risk, providing a more comprehensive understanding of the relationship.

In conclusion, thallium is an independent predictor for diabetes risk; however, thallium may not play a decisive role but is a reference value in predicting diabetes risk among patients with hearing loss.

## Author Contributions

**Conceptualization:** Jing Li.

**Data curation:** Jing Li, Zhi-Gang Lai.

**Formal analysis:** Jing Li.

**Writing—original draft:** Jing Li, Zhi-Gang Lai.

**Methodology:** Zhi-gang Lai, Xiao-Hua Huang.

**Investigation:** Xiao-Hua Huang.

**Supervision:** Xiao-Hua Huang.

**Writing—review and editing:** Xiao-Hua Huang.

## Supplementary Material



## References

[1] Brown CS Emmett SD Robler SK. Global hearing loss prevention. Otolaryngol Clin North Am. 2018;51:575–92. 29525388 10.1016/j.otc.2018.01.006

[2] Samocha-Bonet D Wu B Ryugo DK. Diabetes mellitus and hearing loss: a review. Ageing Res Rev. 2021;71:101423. 34384902 10.1016/j.arr.2021.101423

[3] Orgeta V Mukadam N Sommerlad A. The lancet commission on dementia prevention, intervention, and care: a call for action. Ir J Psychol Med. 2019;36:85–8. 31187723 10.1017/ipm.2018.4

[4] Lin FR Metter EJ O’Brien RJ. Hearing loss and incident dementia. Arch Neurol. 2011;68:214–20. 21320988 10.1001/archneurol.2010.362 PMC3277836

[5] Reed NS Altan A Deal JA. Trends in health care costs and utilization associated with untreated hearing loss over 10 years. JAMA Otolaryngol Head Neck Surg. 2019;145:27–34. 30419131 10.1001/jamaoto.2018.2875 PMC6439810

[6] Genther DJ Frick KD Chen D. Association of hearing loss with hospitalization and burden of disease in older adults. JAMA. 2013;309:2322–4. 23757078 10.1001/jama.2013.5912 PMC3875309

[7] Fellinger J Holzinger D Pollard R. Mental health of deaf people. Lancet. 2012;379:1037–44. 22423884 10.1016/S0140-6736(11)61143-4

[8] Komorowsky CV Brosius FC 3rd Pennathur S. Perspectives on systems biology applications in diabetic kidney disease. J Cardiovasc Transl Res. 2012;5:491–508. 22733404 10.1007/s12265-012-9382-7 PMC3422674

[9] Horikawa C Kodama S Tanaka S. Diabetes and risk of hearing impairment in adults: a meta-analysis. J Clin Endocrinol Metab. 2013;98:51–8. 23150692 10.1210/jc.2012-2119

[10] Olusanya BO Davis AC Hoffman HJ. Hearing loss grades and the International classification of functioning, disability and health. Bull World Health Organ. 2019;97:725–8. 31656340 10.2471/BLT.19.230367 PMC6796665

[11] Leonard A Gerber GB. Mutagenicity, carcinogenicity and teratogenicity of thallium compounds. Mutat Res. 1997;387:47–53. 9254892 10.1016/s1383-5742(97)00022-7

[12] Karbowska B. Presence of thallium in the environment: sources of contaminations, distribution and monitoring methods. Environ Monit Assess. 2016;188:640. 27783348 10.1007/s10661-016-5647-y PMC5080298

[13] Campanella B Onor M D’Ulivo A. Human exposure to thallium through tap water: a study from Valdicastello Carducci and Pietrasanta (Northern Tuscany, Italy). Sci Total Environ. 2016;548-549:33–42. 26799805 10.1016/j.scitotenv.2016.01.010

[14] Campanella B D’Ulivo A Ghezzi L. Influence of environmental and anthropogenic parameters on thallium oxidation state in natural waters. Chemosphere. 2018;196:1–8. 29289846 10.1016/j.chemosphere.2017.12.155

[15] Liu J Luo X Wang J. Thallium contamination in arable soils and vegetables around a steel plant-a newly-found significant source of Tl pollution in South China. Environ Pollut. 2017;224:445–53. 28233568 10.1016/j.envpol.2017.02.025

[16] Wu M Shu Y Song L. Prenatal exposure to thallium is associated with decreased mitochondrial DNA copy number in newborns: evidence from a birth cohort study. Environ Int. 2019;129:470–7. 31158593 10.1016/j.envint.2019.05.053

[17] Di Candia D Muccino E Battistini A. Thallium toxicity due to audultered infusion with thallium sulfate in eight members belonging to the same family nucleus: Autopsy findings and ICP-MS analysis (inductively coupled plasma mass spectrometry) in a triple homicide. Leg Med (Tokyo). 2020;42:101661. 31874453 10.1016/j.legalmed.2019.101661

[18] Li D Yao H Zhu X. Thallium(I) exposure perturbs the gut microbiota and metabolic profile as well as the regional immune function of C57BL/6 J mice. Environ Sci Pollut Res Int. 2022;29:90495–508. 35870064 10.1007/s11356-022-22145-2

[19] Wang X Mukherjee B Park SK. Associations of cumulative exposure to heavy metal mixtures with obesity and its comorbidities among U.S. adults in NHANES 2003-2014. Environ Int. 2018;121(Pt 1):683–94. 30316184 10.1016/j.envint.2018.09.035 PMC6268112

[20] Lee BK Kim Y. Blood cadmium, mercury, and lead and metabolic syndrome in South Korea: 2005-2010 Korean National Health and Nutrition Examination Survey. Am J Ind Med. 2013;56:682–92. 22911659 10.1002/ajim.22107

[21] Chung SM Moon JS Yoon JS. The sex-specific effects of blood lead, mercury, and cadmium levels on hepatic steatosis and fibrosis: Korean nationwide cross-sectional study. J Trace Elem Med Biol. 2020;62:126601. 32634767 10.1016/j.jtemb.2020.126601

[22] Wan H Wang Y Zhang H. Chronic lead exposure induces fatty liver disease associated with the variations of gut microbiota. Ecotoxicol Environ Saf. 2022;232:113257. 35104782 10.1016/j.ecoenv.2022.113257

[23] Frediani JK Naioti EA Vos MB. Arsenic exposure and risk of nonalcoholic fatty liver disease (NAFLD) among U.S. adolescents and adults: an association modified by race/ethnicity, NHANES 2005-2014. Environ Health. 2018;17:6. 29334960 10.1186/s12940-017-0350-1 PMC5769436

[24] Stratakis N Golden-Mason L Margetaki K. In utero exposure to mercury is associated with increased susceptibility to liver injury and inflammation in childhood. Hepatology. 2021;74:1546–59. 33730435 10.1002/hep.31809 PMC8446089

[25] Zou P Li M Chen W. Association between trace metals exposure and hearing loss. Front Public Health. 2022;10:973832. 36062090 10.3389/fpubh.2022.973832 PMC9428401

[26] Hinwood AL Stasinska A Callan AC. Maternal exposure to alkali, alkali earth, transition and other metals: concentrations and predictors of exposure. Environ Pollut. 2015;204:256–63. 25984984 10.1016/j.envpol.2015.04.024

[27] Jain RB. Effect of pregnancy on the levels of urinary metals for females aged 17-39 years old: data from National Health and Nutrition Examination Survey 2003-2010. J Toxicol Environ Health A. 2013;76:86–97. 23294297 10.1080/15287394.2013.738171

[28] Menke A Guallar E Cowie CC. Metals in urine and diabetes in U.S. Adults. Diabetes. 2016;65:164–71. 26542316 10.2337/db15-0316 PMC4686948

[29] Jiang Y Xia W Zhang B. Predictors of thallium exposure and its relation with preterm birth. Environ Pollut. 2018;233:971–6. 29033178 10.1016/j.envpol.2017.09.080

[30] Govarts E Remy S Bruckers L. Combined effects of prenatal exposures to environmental chemicals on birth weight. Int J Environ Res Public Health. 2016;13:495. 27187434 10.3390/ijerph13050495 PMC4881120

[31] Qi J Lai Y Liang C. Prenatal thallium exposure and poor growth in early childhood: a prospective birth cohort study. Environ Int. 2019;123:224–30. 30537637 10.1016/j.envint.2018.12.005

[32] Zhang QQ Li JH Wang YD. Association between maternal thallium exposure and risk of gestational diabetes mellitus: evidence from a birth cohort study. Chemosphere. 2021;270:128637. 33097235 10.1016/j.chemosphere.2020.128637

[33] Li D Li L Yao H. Thallium exposure induces changes in B and T cell generation in mice. Toxicology. 2023;492:153532. 37141935 10.1016/j.tox.2023.153532

[34] Suzuki T Hidaka T Kumagai Y. Environmental pollutants and the immune response. Nat Immunol. 2020;21:1486–95. 33046888 10.1038/s41590-020-0802-6

[35] Ewers U Stiller-Winkler R Idel H. Serum immunoglobulin, complement C3, and salivary IgA levels in lead workers. Environ Res. 1982;29:351–7. 7160352 10.1016/0013-9351(82)90036-6

[36] Winer H Rodrigues GOL Hixon JA. IL-7: comprehensive review. Cytokine. 2022;160:156049. 36201890 10.1016/j.cyto.2022.156049

[37] Osorio-Rico L Santamaria A Galvan-Arzate S. Thallium toxicity: general issues, neurological symptoms, and neurotoxic mechanisms. Adv Neurobiol. 2017;18:345–53. 28889276 10.1007/978-3-319-60189-2_17

[38] Cvjetko P Cvjetko I Pavlica M. Thallium toxicity in humans. Arh Hig Rada Toksikol. 2010;61:111–9. 20338874 10.2478/10004-1254-61-2010-1976

[39] Hanzel CE Verstraeten SV. Thallium induces hydrogen peroxide generation by impairing mitochondrial function. Toxicol Appl Pharmacol. 2006;216:485–92. 16934846 10.1016/j.taap.2006.07.003

[40] Lin DS Huang YW Ho CS. Oxidative insults and mitochondrial DNA mutation promote enhanced autophagy and mitophagy compromising cell viability in pluripotent cell model of mitochondrial disease. Cells. 2019;8:65. 30658448 10.3390/cells8010065 PMC6356288

[41] Maya-Lopez M Mireles-Garcia MV Ramirez-Toledo M. Thallium-induced toxicity in rat brain crude synaptosomal/mitochondrial fractions is sensitive to anti-excitatory and antioxidant agents. Neurotox Res. 2018;33:634–40. 29313218 10.1007/s12640-017-9863-1

[42] Rosa MJ Just AC Guerra MS. Identifying sensitive windows for prenatal particulate air pollution exposure and mitochondrial DNA content in cord blood. Environ Int. 2017;98:198–203. 27843010 10.1016/j.envint.2016.11.007 PMC5139686

[43] Yang K Forman MR Graham BH. Association between pre-diagnostic leukocyte mitochondrial DNA copy number and survival among colorectal cancer patients. Cancer Epidemiol. 2020;68:101778. 32674053 10.1016/j.canep.2020.101778 PMC8981089

[44] Oppong RF Terracciano A Picard M. Personality traits are consistently associated with blood mitochondrial DNA copy number estimated from genome sequences in two genetic cohort studies. Elife. 2022;11:e77806. 36537669 10.7554/eLife.77806 PMC9767459

[45] Ashar FN Zhang Y Longchamps RJ. Association of mitochondrial DNA copy number with cardiovascular disease. JAMA Cardiol. 2017;2:1247–55. 29049454 10.1001/jamacardio.2017.3683 PMC5710361

