# Supplementary figures and images for: Association of Thallium with Diabetes Risk among Patients with Hearing Loss: Result from NHANES 2013 to 2018
Source: Medicine (Baltimore). 2024 Mar 1;103(9):e37317. doi: 10.1097/MD.0000000000037317 (PMC10906567; doi:10.1097/MD.0000000000037317)

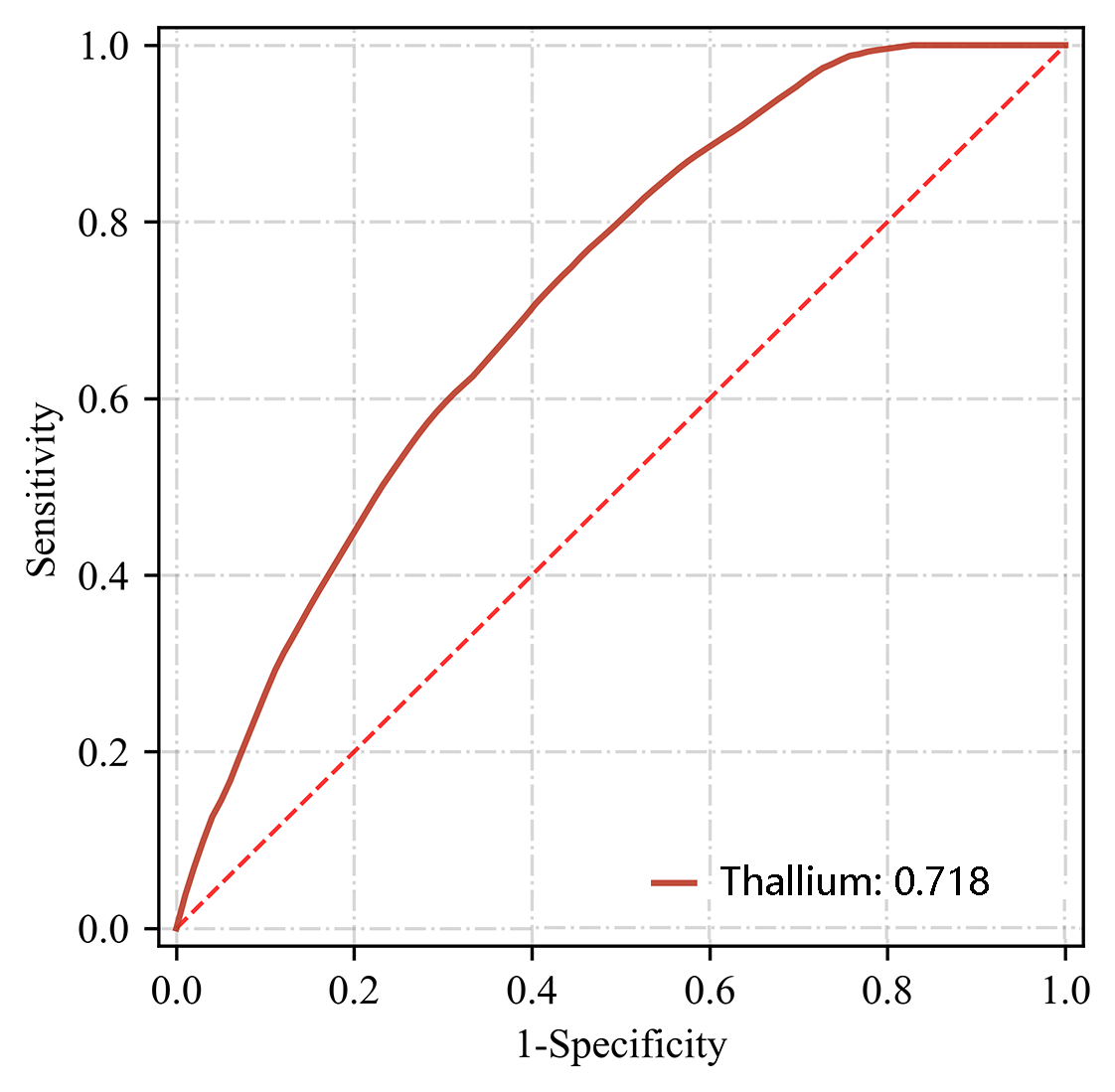

Supplement: Supplementary file 1 [file medi-103-e37317-s001.tif]
